# The impact of COVID-19 on the livelihoods of Kenyan slum dwellers and the need for an integrated policy approach

**DOI:** 10.1371/journal.pone.0271196

**Published:** 2022-08-02

**Authors:** Daniel Solymári, Edward Kairu, Ráhel Czirják, István Tarrósy

**Affiliations:** 1 Political Science Doctoral Programme, Interdisciplinary Doctoral School, University of Pécs, Pécs, Hungary; 2 St. Paul’s University, Limuru, Kenya; 3 Principal Consultant, ETC Consulting Limited, Nairobi, Kenya; 4 Earth Sciences Doctoral School, University of Pécs, Pécs, Hungary; 5 Department of Political Science and International Studies, Faculty of Humanities and Social Sciences, University of Pécs, Pécs, Hungary; University of Georgia, UNITED STATES

## Abstract

This paper aims to deal with the impact of COVID-19 on the livelihoods of disadvantaged persons living in slums in Kenya. Months after the first case of COVID-19 was reported in Kenya in March 2020, most of the studies that have been carried out pertaining to its impact on slum dwellers have concentrated on narrowly defined concerns e.g. the impact of COVID-19 on youth, gender based violence and nutrition. It is thus difficult to get a clear global picture of the overall impact of COVID-19 on the livelihoods of slum dwellers in Kenya. This paper relies on information gathered during a comprehensive qualitative micro study covering numerous aspects of slum dwellers’ livelihoods, as well as information that has been produced by the Ministry of Health, civil society organizations that work in specific slums, private research organizations as well as local and foreign media houses. The slums whose information is reported in this paper were selected to be indicative of the over 300 slums that are located in Nairobi and Mombasa, the two most important cities in Kenya. The analysis concludes that slum dwellers were potentially at a higher risk under the pressures of COVID-19 of deteriorating conditions with regard to the provision of health services, employment, gender-based violence, education and youth-related problems, and human rights violations, and offers several recommendations to the government.

## Introduction

**Fig 1:**
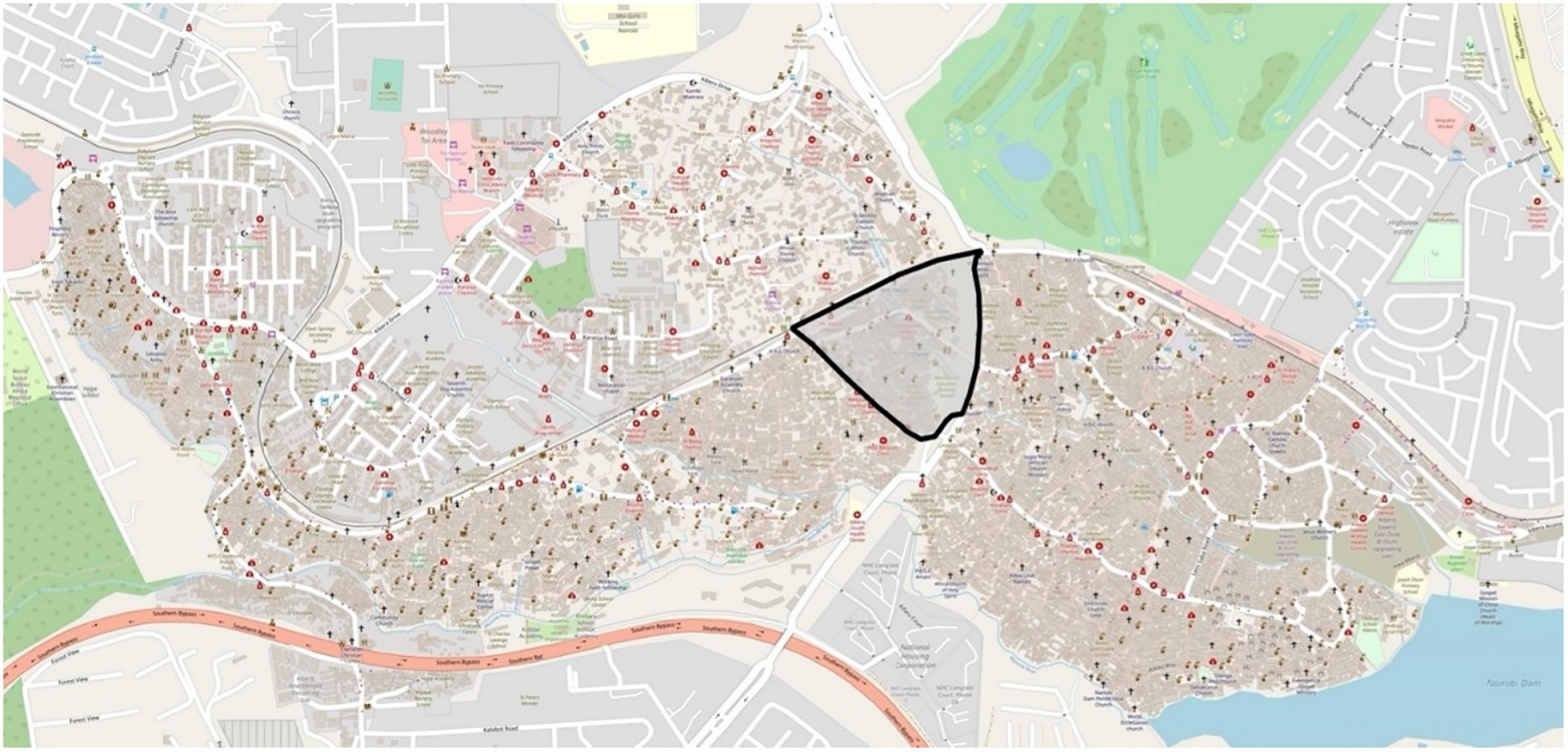
The catchment area of the research within Kibera.

**Fig 2:**
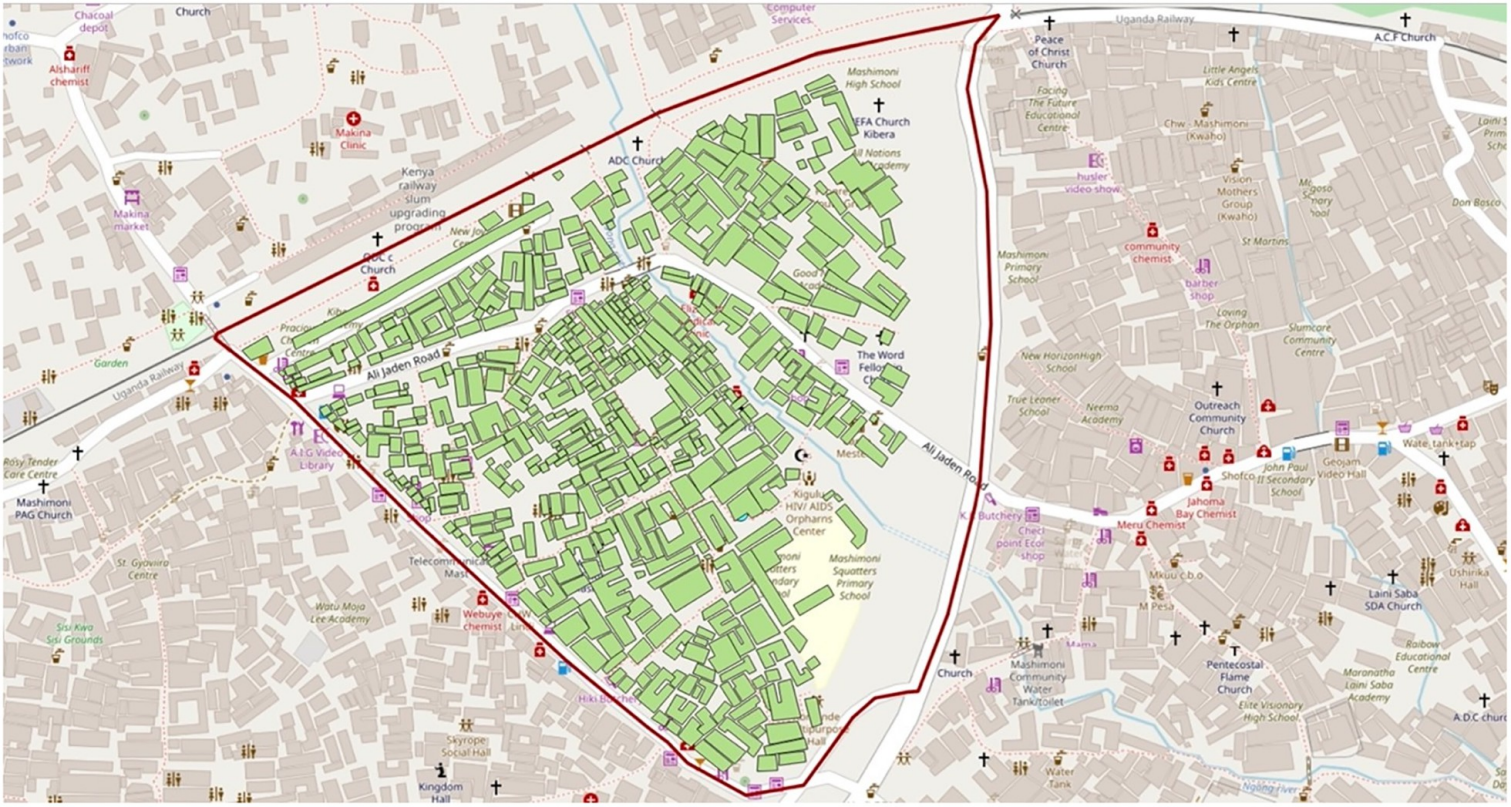
The focal triangle of the research.

Well over two years after the first case of COVID-19 was reported in Kenya in March 2020, most of the studies that have been carried out pertaining to its impact on slum dwellers have concentrated on narrowly defined concerns e.g. the impact of COVID-19 on youth, gender based violence and nutrition. This paper sets the goal to present a holistic view of the impact of COVID-19 on the livelihoods of disadvantaged persons living in the slums of Kenya’s large urban settlements. It relies on information gathered during a comprehensive qualitative micro study covering health, psychosocial, hygiene, education and gender aspects of slum dwellers’ livelihoods led by the Hungarian Charity Service of the Order of Malta and the Africa Research Centre of the University of Pécs, from the beginning of 2021 until summer the same year. It is also based on information that has been produced by the Ministry of Health, civil society organizations that work in specific slums, private research organizations as well as local and foreign printed and online media houses (with particular attention to Kenyan press) examined during the mentioned period of time. In addition to the desk survey that was conducted, open ended questionnaires were used to collect primary data by means of face-to-face interviews with 50 key informants, in most cases the chairpersons of the Community Based Organizations that operate in fifteen slum-villages located in the cities of Nairobi and Mombasa (Figs 1 and 2).

Our holistic approach can resonate well with the policy framework the Nairobi-based UN-Habitat has also been advocating for. In April 2016, Executive Director Joan Clos underscored for Africa Renewal that development must be accelerated and slums “should be integrated in a much better manner [as] you cannot fight slums in an isolated manner. What you need is an integrated policy that addresses the livelihoods of people, such as employment, income, training and human capital” [1]. In addition to housing challenges, cities in general are confronted with many other demands such as climate change, as well as man-made disasters in the form of armed conflicts, civil wars and the like, in different corners of the African continent. While the rapid urbanization process results in megacities also in this part of the world, which “will sprawl and smaller cities and towns will grow into economic powerhouses […] inequalities between social classes and ethnic groups are deepening” [2]. There are numerous urbanization pressures and policymakers in many cases cannot find the right solutions, as they struggle with thinking in a cross-sectoral, holistic way. Nairobi, as a matter of fact, offers an interesting example for “openings in governance to provide for popular participation and more effective service delivery” [3], which if we translate to areas of segregation, sheds light on how slums are “likely to reduce the urban quality of life even for those individuals not living in them” [4]. However, Pieterse is right, as in the majority of African urban settlements, “slum life is the norm” [5] (for many). The COVID-19 pandemic produced negative consequences for the population of these informal urban settlements, further deepened the marginalization of slum dwellers, and challenged urban policymakers, as these segregated people are the most vulnerable strata of Kenyan society, consequently they are extremely exposed to any social and structural challenges, such as a pandemic. Due to their vulnerability, they alone are ill-equipped to deal effectively with such challenges.

In the present paper, we consequently focus on the needs of people in urban slums for which years of research and active engagement in lending assistance are at hand. Our overall goal is to gain a more accurate understanding of the daily struggle of slum dwellers in their years during the different phases of Covid-19 pandemic and thus of a more holistic view of the impact of the pandemic to Kenyan urban “slum society”.

With a focus on slums in Nairobi and Mombasa, this study operated with the following assumptions that

- the Government of Kenya acknowledges the existence of slums most of which are illegally located on government land;
- the Government of Kenya values all its citizens equally and that the government has a cardinal responsibility of protecting the lives of slum dwellers;
- and lastly that the Government of Kenya as well as its development partners and other stakeholders such as local and international non-state actors would value holistic information on the impact of COVID-19 on the livelihoods of slum dwellers in Kenya.

We aim at presenting the results of this novel fieldwork, via which we can complement the complex and multifactorial picture that the pandemic has shaped in Kenya. Our intention is to draw attention to one of the most underrepresented areas of the ongoing global crisis, the challenges posed by the coronavirus epidemic in many of Sub-Saharan African economically disadvantaged metropolitan areas.

### Brief chronological summary of the unfolding of COVID-19 events in Kenya

Since COVID-19 has in one way or another affected virtually the entire population in Kenya, it is relatively easy for the Government of Kenya and its development allies, to overlook the interests of slum dwellers. At the same time, development of holistic remedial measures by the Government of Kenya and other stakeholders has been problematic, in part due to the segmented approach of documenting the impact of COVID-19. On 12^th^ January 2020, the World Health Organization (WHO) confirmed that a novel coronavirus was the cause of a respiratory illness in a cluster of people in Wuhan City [6], Hubei Province, China, which was reported to the WHO on 31^st^ December 2019. On 13^th^ March 2020 [7], the first COVID-19 case was recorded in Kenya, which involved a 27-year-old Kenyan woman who had traveled to Kenya from the USA via London [8]. The Kenyan government identified and isolated a number of people who had come into contact with this first case. On 15^th^ March 2020, Kenya’s Cabinet Secretary for Health, Mutahi Kagwe, announced that two people who had sat next to the initial patient on the aircraft in transit from the United States had also tested positive for COVID-19. On 22^nd^ March 2020, following the confirmation of additional eight cases, bringing the total to 16 nationally, the Kenyan government became more vigilant about putting in place suitable mitigation measures against COVID-19 [9].

According to Jason Corburn et al. [10], the Government of Kenya took the following measures to protect residents of urban informal settlements, the homeless and those living in precarious settlements from the negative impacts of COVID-19:

- Apply an immediate moratorium on evictions;
- Institute informal settlements/slum emergency planning committees in every urban informal settlement;
- Provide an immediate guarantee of payments to the poor;
- Immediately train and deploy community health workers;
- Immediately meet Sphere Humanitarian standards for water, sanitation, and hygiene;
- Provide immediate food assistance;
- Develop and implement a solid waste collection strategy; and
- Implement immediately a plan for mobility and health care.

By June 2021, three waves of COVID-19 had been experienced in Kenya with a possible fourth wave expected to hit the country by mid-2021. Sequentially, the COVID-19 waves occurred as follows: first COVID-19 wave between March and September 2020; second wave between October and December 2020; third between March and June 2021 and fourth wave from July 2021. During the first wave, the pandemic was mainly concentrated in the capital Nairobi and Mombasa, the tourism bedrock at the Coast. Restriction of movement in and out of the two largest cities in Kenya helped cushion rural areas where most residents continued to ignore health measures that include social distancing and the wearing of face masks [11].

On 29^th^ October 2020, Mutahi Kagwe, Kenya’s cabinet secretary for health, noted that the country was experiencing a second wave of COVID-19 infections. He stated that “We have a second wave of the disease in the country and for us the tough balance is now between keeping our economy running and keeping people safe. This is creating challenges” [12].

On 10^th^ March 2021, Li Hualing writing for Xinhua newspaper quoted the Ministry of Health as alerting Kenyans to maintain their vigilance since the country was firmly in its third wave of COVID-19 pandemic, due to the introduction of Alpha and Beta variants of SARS-CoV-2 virus [13]. Three months later, according to Magdalene Saya [14] (writing for the Star newspaper on 1^st^ July 2021), Kenyan government experts warned that Kenya was likely to experience a fourth COVID-19 wave from mid-July fueled by the highly infectious Delta variant [14]. The highly transmissible variant which was first detected in India was gaining dominance over the Alpha variant (which had characterized the third wave in Kenya especially in the Western and Nyanza regions). Kenyan government experts projected the start of a fourth wave to be imminent in the Coastal region and Nairobi County, which was already causing a surge in Nyanza and Western regions. Once again, Ministry of Health was quoted as saying that “The timing of the next (fourth) wave is imminent for the country as a whole or, while in the case of the lakeside region it is currently occurring” [14].

According to the Center for Control of Diseases and Prevention (CDC), WHO and the European Centre for Disease Prevention and Control (ECDC), as of 21^st^ September 2021, Kenya had confirmed 246,956 cases of COVID-19, with 5,008 deaths and 238,448 recoveries. Mass vaccination commenced on 5^th^ March 2021, initially with 1.02 million doses of AstraZeneca’s Covishield vaccine supplied under the COVAX pillar. The second dose vaccination began on 28^th^ May 2021 [15].

According to the Ministry of Health, by 20^th^ September 2021, out of 5,358,220 vaccines in stock, a total of 3,409,017 vaccines (Astra-Zeneca, Moderna, Johnson and Johnson and Pfizer, with plans said to be underway to also roll out Sinopharm in the near future) had been administered across the country, with 2,543,876 being first doses and 865,141 being second doses. The uptake of the second dose among those who received their first dose was at 34.0% while the proportion of adults who are fully vaccinated was 3.2%. The Government of Kenya has been working towards vaccinating a target population of 10 million persons by the end of 2021. The Ministry of Health further stated that the single-shot Johnson and Johnson vaccine is expected to be of great utility particularly when reaching the primary healthcare level of the country’s health system where the uptake of the vaccine continues to be low [15].

In order for the Government of Kenya to vaccinate all its population, the country has been relying on vaccine donations from its development allies. For instance, on 23^th^ August 2021, Kenya received 880,460 doses of the Moderna COVID-19 vaccines from the United States of America, which formed the first of two shipments totaling 1.76 million doses. Similarly, on 4^th^ September 2021, Kenya received 141,600 doses of the first consignment of the Johnson and Johnson vaccine donated by the United States of America [16].

## Methodology

In our research we mainly analysed secondary sources, in addition to which chose interview as the primary means of data collection. Our interviewees were residents of the visited Kenyan slums. Based upon the required ethics approvals, as well as consent to participate procedure, the interviews were conducted with the help of local assistants (local leaders, social workers, co-workers of NGOs, etc.) who were familiar with the respective informal urban settlement.

Personal participation in connection with the study happened on a voluntary basis. Data collection and analysis of the gathered surveys were strictly anonymized. The ethical approval of the study was designed as per (1) the Humanitarian Charter of the Sphere Project and Handbook in line with the CHS Humanitarian Minimum Standards, (2) the Code of Conduct of the Hungarian Charity Service of the Order of Malta and its CEO and (3) the Scientific Research Ethics Committee of the Medical Research Council of Hungary. The latter one is based on the Codex of Ethics of Scientific Knowledge of the Hungarian Academy of Sciences. To ensure the ethical aspects and the principle of homogeneity, the questionnaires were led and supervised by the same social worker and research interviewer in each given interview. All interviewers received the same instructions before administering the questionnaires. Influencing respondents and qualifying their responses were strictly forbidden. Minors were not included in this study.

The research was part of a development aid project evaluation, consequently under the Kenyan Science Technology and Innovation (Research Licensing) Regulation 2014, no permission was required from the National Commission for Science and Technology and Innovation, as local IRB. However, voluntarily, and freely the study had been reported verbally to the State Department of Housing and Urban Development in Nairobi, which noted and approved it verbally. As it was a voluntary notification, no documentation nor recording was required, therefore written consent was not obtained. Additional information regarding the ethical, cultural, and scientific considerations specific to inclusivity in global research is included in the S2 File.

As the first step of the primary data collection, we selected the major slums within the territories of Nairobi (Kibera and Mathare, which are rather similar) and Mombasa (Bangladesh) as a completely different case, which was used as a possible counterexample in the survey, or rather an out-of-sample testing. These became the data collection sites from which the sample data were received. Even though this survey is non-representative, and does not allow us to draw general conclusions, it still yields important and fresh knowledge from the field helping to create a more comprehensive picture, in order to better understand several of the connecting details in a more effective way. We are aware that the selected slums are only just a few of the hundreds, but they possess most characteristics. A survey such as ours in the micro study we had designed in these slums is feasible, in particular with the involvement of the local community leaders who are familiar with such scientific initiatives. There was a guided element in this step of the sampling: Kibera was automatically and intentionally included in the data collection areas (not as a result of random selection), as we were already present here with other field data collection activities, thus we were able to conduct the interviews ourselves without the involvement of an external facilitator.

As a next step, we contacted the government and local NGO actors active in these slums and requested their assistance in conducting the interviews. After lengthy considerations, we agreed upon interviews as the primary means for collecting information. It would have been interesting to conduct a multi-question questionnaire based on a greater number of samples in all the data collection areas, however, due to our finite resources, we were forced to prioritize which aspects of the study should be given advantage. Weighing the advantages and disadvantages, we decided that in the case of this study, comparability and information from as many areas as possible are the most important aspects, therefore we worked with fewer items and fewer questions instead. In return, we opted for an interview that better matched these parameters. Due to the small number of items, the quantitative processing of the questionnaire would not have been too meaningful anyway. Due to the less limiting nature of the methodology during the interviews we had the opportunity to collect information that otherwise we would not have had the chance to do using questionnaires with few questions.

After having succeeded to arrange an interviewer in all areas of data collection (thanks again to them for assisting us voluntarily, sacrificing their free time) and with the help of the available data sources and estimates, we determined the number of interviews we needed to collect from a given data collection area to arrive at a roughly proportionate demographic numerical sample. A total of 50 interviews were conducted by our assistants. The intention of our paper is not to accurately reconstruct the antecedents and events of the pandemic or to give a detailed narration of its historical unfolding. Instead, it is directed at describing the local slum dwellers’ system of relations and to use this information to both better understand the sentiments of the urban poor and in doing so, guide aid efforts.

**Fig 3:**
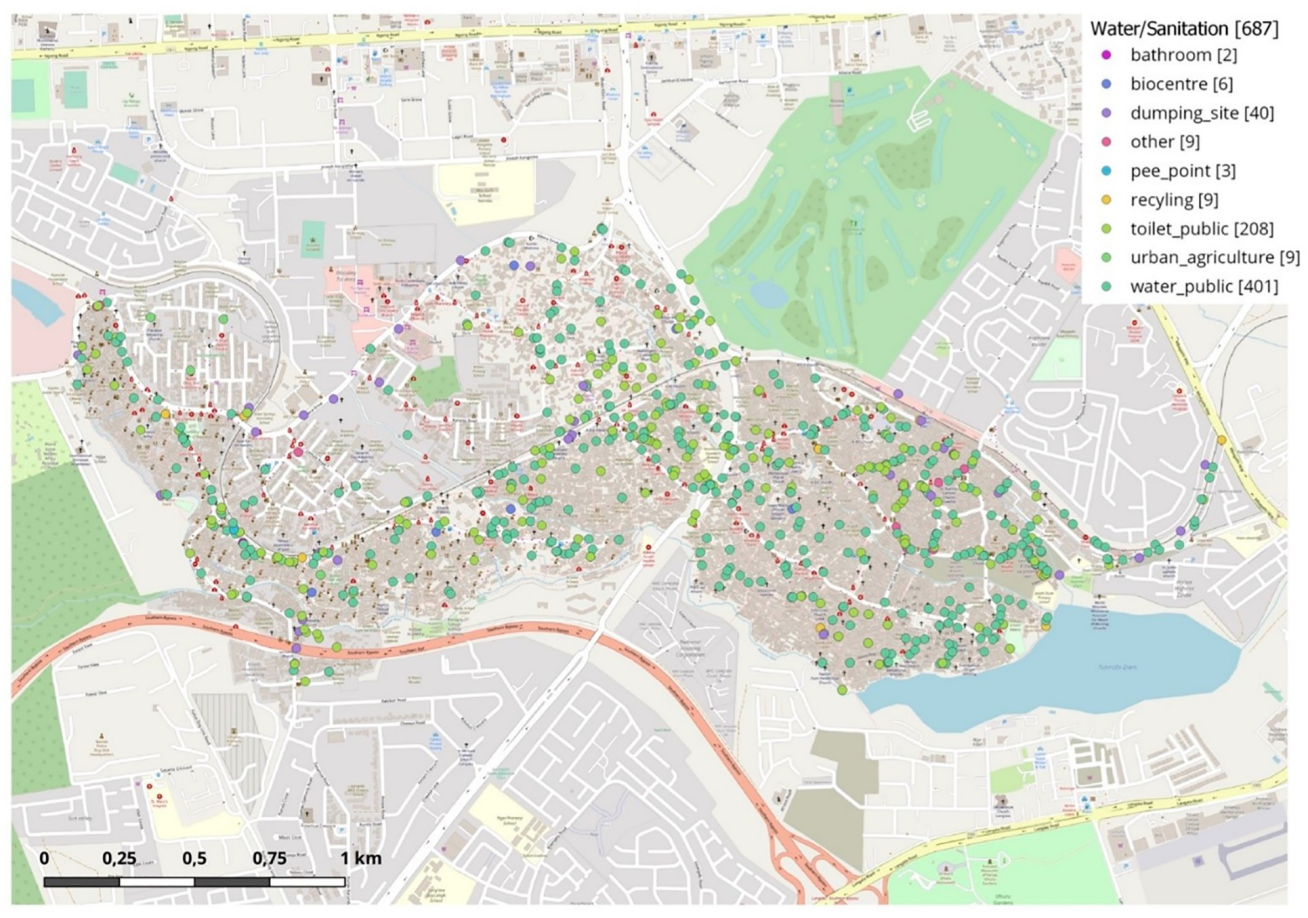
Categories of water and sanitation facilities in the slum—Own edition.

When we determined the scope of the research, we had to take into account our capacities. The sample size was determined in 50 in-depth interviews. This was followed by the exact geographical determination of the research area. As there are many sanitation facilities within Kibera, Kambi Muru village, and we were interested in the effects of the one that the Hungarian Charity Service of the Order of Malta built in April 2012, we wanted to conduct the research within the direct agglomeration/catchment area of the facility. Fig 3 shows the water and sanitation facilities within the slum area. The catchment area for our research was delimited by Voronoi polygon, but it would have been too small for the one third of the 50 interviews, therefore, the study area was expanded to include a nearly triangular, 44,000 m^2^-area, well-defined by road. The locations of interviews were defined by random sampling. Based on the open source database of Open Street Map about the buildings there, 17 buildings were randomly picked. In case of the chosen buildings are not residential houses, 4 backup buildings were randomly chosen as well. Our maps were drawn accordingly.

In the case of the other two slums apart from Kibera, interviewers were asked to find a busy point within the given slum where a significant portion of the slum’s residents passed through (public transport hubs, markets, major intersections, etc.). The interviews were conducted at these sites, and the interviews in the data collection area were divided into three (roughly) equal parts and were conducted starting at 8 a.m., noon, and 4 p.m. during a weekday, therefore the data were collected at each site approximately at the same time. Interviewees were selected by choosing every tenth pedestrian, beginning at the specified time, then asking if he or she would participate in the data collection. Whether the answer was negative or affirmative, after parting with the (potential) interviewee, the interviewer again started counting the pedestrians and again asked the tenth person for an interview, continuing so until the quota for the current time band was filled. This type of sampling ensured that the interviewer could influence as little as possible who the selected interviewee was, however, the disadvantage of this approach is that those who are not typically on the streets in the given time zone (those with illnesses, working different hours e.g. at night, etc.) are underrepresented.

Completing an interview took approximately 15–30 minutes, the interview itself was guided by the questions pre-supplied but essentially it was a conversation of informal character. One topic was generally covered by one (in some cases two) generic question, but in the methodological guide we gave the interviewer the opportunity to ask additional supplementary questions if he or she deemed that a given topic was more relevant to the interviewee.

The interviews were recorded and sent to us by the interviewers, and the processing was carried out by us, anonymizing the interviews as a first step. During the course of the interview analysis, we then looked for the common features emerging from the answers and also recorded any outstanding, individual characteristics.

In the interview, we asked questions with regard to the following topics:

- The demographic background of the interviewee, those living with him/her in the same household
- Workplace, job opportunities, unemployment, loss of income
- Living space (the narrowing of it), moving, leaving home
- Shopping options, stockpiling supplies, changes in consumption patterns
- Changes in family and community life, the emergence of deviant forms of behaviour
- Transportation options
- Epidemiology: access to information
- Epidemiology: health measures (e.g. hand washing, use of mask, testing, vaccination, etc.)
- Epidemiology: unexpected, disproportionate measures by the authorities

In the following sections we are presenting the impacts of COVID-19 on several activities and issues in the targeted informal urban settlement sites. Our survey carried indicative values, as it was capable to look at restrictions, containment, the provision of health services, employment and income generation, gender-based violence and gender inequalities, education for slum dwellers’ children, youth-related problems, and human rights violations. We intentionally embedded the knowledge we had obtained from the ground throughout all the different sections of the paper dealing with the above major challenges, and do not separate a designated section for the research results.

### Curfews, lockdowns, and containment on the livelihoods of slums dwellers in Kenya

In response to the COVID-19 pandemic which hit Kenya in March 2020, citizens were instructed to observe stringent mitigation measures including social distancing, frequent handwashing, use of hand-sanitizers and avoiding huge gatherings. These measures were accompanied by a nationwide night time curfew, closure of non-essential services, learning institutions and places of worship, a stricter regulation of the public transport sector and cessation of movement in areas with a high number of positive COVID-19 cases. In retrospect, it is clear that some of these measures particularly curfews, lockdowns and containment, have highlighted pre-existing deep social inequalities in Kenya which have resulted in many slum dwellers being pushed further into poverty [17]. In many ways, the experience of COVID-19 in Kenya can be equated to a story of poverty.

COVID-19 containment measures led to massive closure of companies and other businesses leading to far reaching effects especially for low income daily wage earners, many of whom live in slums. The loss of income also affected the small scale traders in the slums due to low purchasing power as a result of the surge in the number of unemployed persons in the slums. Many households which were already poverty stricken have been rendered even more vulnerable and are unable to afford food and to pay rent. In most urban slums, a sizable percentage of the residents initially vacated the city for the countryside to flee from COVID-19 [17].

According to Israel Nyaburi Nyadera and Francis Onditi [18], “as the virus continues to spread rapidly, the more popular measures such as curfews, lockdown, and working from home have overlooked the fate of slum dwellers, who are left to choose between life and livelihood”.

On 27^th^ March 2020, in Mombasa, more than 2 hours before curfew took effect, police teargassed crowds of people lining up to board a ferry back home from work, beating them with batons and gun butts, kicking, slapping, and forcing them to huddle together or lie on top of each other [19]. On 31^st^ March 2020, in Kiamaiko, an informal settlement located in Nairobi, a 13-year-old boy was shot dead, allegedly by police, on the balcony of his family home, 20 minutes after the curfew had commenced [20]. According to Human Rights Watch [21], at least six people died from police violence during the first 10 days of Kenya dusk-to-dawn curfew, imposed on March 27^th^ 2020 to contain the spread of COVID-19. The police, without apparent justification, shot and beat people at markets or returning home from work, even before the daily start of the curfew. According to the key informants who were interviewed in Nairobi and Mombasa slums, COVID-19 related curfews, lockdowns, and containment have had different impacts on the livelihoods of slum dwellers in Kenya. Regrettably, a disproportionate amount of the attendant violence in the early phase of COVID-19 in Kenya was mainly directed to Kenyans living in slums, where cases of police brutality were already commonplace. For instance, the 7pm– 5am curfew that was announced by the Government of Kenya on 25^th^ March 2020 [22] was accompanied by reports of police brutality with the worst reports emanating from Mombasa County, where one person died at the Likoni ferry. As Jason Corburn et al. stated [10] “the political and economic shocks and instability that are happening now and are likely to follow beyond the current COVID-19 epidemic, will possibly kill more and lead to more disability than the coronavirus itself”. Corburn et al. [10] further state that “Government-enforced social isolation may help relatively affluent populations limit the spread of COVID-19, but these measures can be devastating for the nearly 1 billion people around the globe currently dwelling in urban slums, where physical space is scarce, and many rely on daily wage labor for survival”.

Unfortunately, detention of slum dwellers by police resulted in crowding of people into small spaces, contrary to the curfew’s goal of increasing social distancing. According to the key informants who were interviewed in Nairobi slums, from the onset when Kenyans were asked to observe social distancing, many of them took this to mean isolation and staying away from others. They shunned those who caught the virus and declared them as outcasts. Accordingly, social distancing, particularly from those affected with the disease, was so emphasized by the Government of Kenya that it led to massive stigmatization and stereotyping. With the onset of COVID-19 and its attendant mitigation measures that were prescribed by the government, many Kenyans rushed to the supermarkets and stores to stock up on sanitizers and essential foodstuffs to see them through the anticipated period of social distancing. However, slum dwellers who did not have high purchasing power as well as limited refrigeration facilities found it hard to engage in the panic buying.

The situation whereby many slum dwellers kept away from social life, disintegrated the social fabric of communities and saw many slum dwellers and their nuclear families becoming islands on their own. Elsewhere in the country, closure of recreational centres, social and entertainment joints widened the rift between people. Shutting of mosques and churches and other places of worship further exacerbated the situation of Kenyan citizens. The resultant social distancing has led to social disintegration which is currently linked to issues such as increased radicalization, gender-based violence, early pregnancies, teenage delinquency and high rates of suicide during the COVID-19 pandemic. As a result of COVID-19, what was before a social Kenyan way of life has degenerated into an individualistic society where keeping away from others is the new norm. However, this is difficult in slums where the high population densities dictate that people live in close proximity.

Additionally, the current measures to restrict spread of COVID-19 has had a direct negative impact on the livelihoods of tens of thousands of urban slum dwellers across Kenya. Generally, slum dwellers live in crowded single-roomed shanties where a number of households share bathrooms, sinks, and water points. There is little or no space for children to play and social distancing is impossible. They also do not have personal/family means of transport and so many of them use crowded public transport, which includes the use of motor bikes that sometimes carry up to three passengers on a single bike. Unfortunately, blanket containment measures that have been imposed by the government Kenya to control the coronavirus pandemic have denied poor slum dwellers access to sufficient nutritious food and livelihoods. This is according to early findings from an ongoing evidence-based study to assess the impact of COVID-19 on dietary patterns among households in Nairobi’s informal settlements cited by Africa.com [23]. To complicate matters further, it is important to note that initially, due to misinformation, many slum residents believed that COVID-19 would only affect the affluent communities in Kenya as well as believing that Africans were safe from COVID-19.

Given the public outcry that followed the police brutality, a petition was filed by the Law Society of Kenya insisting that the curfew was unconstitutional, mainly because “it was blank and indefinite, and because it contravened the Public Order Act” and that the curfew posed a threat to the health of the general Kenyan population. The petition further asserted that, “the police recklessly hounded large crowds of people on the ground, contrary to WHO advice on social distancing”. In response to this petition, the first respondent (Kenyan police) stopped the media from monitoring their movement and assaulted a number of journalists covering the curfew enforcement process. On 30^th^ March 2020, the High Court of Kenya upheld the curfew itself, but barred police from using excessive force to enforce the curfew and demanded the police provide guidelines for observing the curfew [24].

During the early phases of COVID-19 pandemic in Kenya, it was generally thought that if coronavirus hits the slums, it would spread like wildfire. For instance, in April 2020, the Ministry of Health warned that Kibera slum was emerging as a coronavirus hotspot [25]. Thereafter, the Government of Kenya started viewing informal settlements are the weakest link in efforts to tame COVID-19. It was believed that because of their unplanned nature, informal settlements are filthy, crowded, lack basic amenities and proper housing. However, as of June 2021, that cataclysmic prediction has not manifested itself. This is due to the following reasons:

- Enhanced surveillance work by the Ministry of Health in the major slums particularly in Kibera slum.
- Accelerated mass testing targeting the informal settlement which are one of the high-risk areas in the country.
- The slums social fabric which has for a long time been considered an opportunity for key interventions e.g. COVID-19;
- Residents of informal settlements are used to preventing and managing infectious diseases, like tuberculosis, cholera, typhoid, pneumonia, and HIV/AIDS. Nonetheless, COVID-19 has ended up presenting new challenges for these residents;
- People living in informal settlements have much more contacts with each other than those living in the formal settlements in the major cities of Kenya;
- Challenges of disaggregating national data to highlight the information for informal settlements;
- Provision of sufficient water and hygiene interventions in slum areas by the relevant regional agencies e.g. Nairobi Metropolitan Services (NMS), together with Athi Water Works Development Agency have done a complete community water project that is independent and does not depend on the normal water supply in Nairobi County. In the case of Huruma informal settlement, water has played a major role during this COVID-19 pandemic since the residents have continued to enjoying a constant flow of clean water.

### WASH-related COVID-19 governmental directives

For a variety of reasons, many slum dwellers in Kenya have not been able to observe the main government directives including staying at home, using face masks, sanitizing, observing social distancing, self-isolating for those afflicted by COVID-19, to the degree that was required by the government. For instance, slum dwellers would prefer to save the available water for making food and drinking as opposed to using the water to wash their hands frequently, as per COVID-19 government directives. In most settlements in Nairobi, the cost of water per 20 litre jerrycan increased significantly after the confirmation of the first COVID-19 case in Kenya—doubling in many communities, thereby making it harder for slum dwellers to spend their limited funds to purchase for hand washing.

Historically, there has always been an acute water shortage in most slums in Kenya. However, that reality notwithstanding, in the case of Nairobi slums, the national government has recently installed elevated free-water tanks in high density informal areas. Athi Water Works Development Agency says it has pumped 14 million litres of water into the slums [26]. Nairobi Metropolitan Services has sunk 93 boreholes complete with elevated water tanks, including 12 in Kibera, 10 in Mukuru and eight in Mathare slums [26]. In most settlements in Nairobi, the cost of water per 20 litre jerrycan increased significantly after the confirmation of the first COVID-19 case in Kenya—doubling in many communities. It is ironic that COVID-19 provided the jolt that enabled the Kenyan government authorities to provide slum areas with free community water and sanitation services. As of June 2021, 750,000 people from the Nairobi informal settlement areas benefited from this community water project noting that they had access to clean reliable water supply. This was courtesy of the Nairobi Metropolitan Services (NMS), together with Athi Water Works Development Agency, both of which have implemented complete community water projects in the informal settlements of Nairobi [27].

Regarding to sanitation in Kenya’s slums, national and county governments have cooperated with a view to providing slum dwellers with viable solutions. For instance, Athi Water Works Development Agency has constructed 15 ablution blocks in Kibera with residents now having a more hygienic way to dispose of solid waste [27]. The ablution blocks are also connected to sewer line. This was aimed at minimizing the incidence of the famous ‘flying toilets’ that have been a menace and a threat to general hygiene of the informal settlement across Kenya. Since the outbreak of COVID-19 in Kenya, the cost of public toilets in communities had generally remained the same, with exceptional pockets of increases in some settlements. In the slums which have not benefitted from national government WASH interventions, independent water vendors have filled the gap, with handcarts and tricycle pick-ups carrying 20-litre jerry cans being highly predominant. In the slums where the government has been providing free water to slum dwellers, COVID-19 can be said to have reduced the water vending business in slums drastically. Suddenly, water vendors who used to make a killing before the entry of COVID-19 are competing with water tankers from the Nairobi Metropolitan Services (NMS) and organizations such as Shining Hope for Communities (Shofco), Safaricom Foundation and Kibera Town Centre.

With regard to the uptake of handwashing, use of face masks and sanitizers, and the practice of social distancing, the following observations apply to the slum dwellers in Kenya:

- Most slum dwellers use one disposable mask for more than a week, essentially not for protection against COVID-19 infection, but for avoidance of the wrath of law enforcers who have repeatedly used this offence as an excuse for extorting money from the slum dwellers. However, considering the difficult economic situation, many complain that the cost of masks is too high (Ksh 20 to 50). They are forced to make sacrifices to follow this directive. The biggest concern from a public health perspective is the improper use and disposal of masks. Some keep them in their pocket while others are hanging them on their necks, others covering the chin while others are holding them in their hands.
- The majority of slum communities, sanitizers are viewed as a luxury item;
- Slum residents have been trying their best to practice personal protective measures especially handwashing. At the onset of COVID-19 in early 2020, local shops and small traders set up simple handwashing stations next to their petty businesses for their customers. The local leadership has helped to mobilize partners to set up handwashing stations in busy areas such as local markets and boda (motorcycle) terminus. The main challenges with handwashing was the inconsistent supply of water due to rationing and lack of soap.
- Adhering to social distancing has been a major challenge for most slum communities in Kenya. This can be attributed to congestion within homes and houses being very close to each other and the fact that slums are also more communal and there is little private space.

### Provision of health services

According to the United Nations [28], about 1 billion persons live in slums globally. Numerous studies have shown that this population is particularly vulnerable to infectious diseases. According to Samuel Muhula et al. [29], “People living in resource-limited settings such as informal settlements, and women and children, are particularly vulnerable during pandemics and experience a relatively higher disease burden associated with the pandemic”. The above-cited reality is particularly true in Kenya, where the biggest health challenge is that a large proportion of slum dwellers has underlying health conditions such as respiratory bacterial infections (e.g. tuberculosis) and other chronic diseases related to nutrition and poor health, HIV, TB; non-communicable diseases like hypertension and diabetes all of which have enhanced the risk of slum dwellers developing severe COVID-19 symptoms. Some of the viruses of most concern in Kenya’s informal settlements are those that cause diarrhea and pneumonia.

According to Christine Musyimi et al. [30], “People in slum areas in Kenya may be at a higher risk of contracting COVID-19 due to overcrowding, limited access to running water and employment opportunities to cater for their needs”. For slum dwellers, government restriction measures also have had detrimental effects on the mental health and socio-economic aspects of individuals, care givers and communities. According to Government of Kenya and the Inter-parliamentary Union [31], “Pneumonia, diarrhea and malaria remain the leading cause of child mortality in Kenya”. As Samuel Muhula et al. [29] stated, “People living in resource-limited settings such as informal settlements, and women and children, are particularly vulnerable during pandemics and experience a relatively higher disease burden associated with the pandemic”. To compound this vulnerability for Kenyans living in informal settlements, is the observation that the current and previous health schemes did not fail due to the lack of resources, but due to corruption and poor strategies thereby making millions of citizens miss out on this important program. Very few slum dwellers in Kenya are covered by the national health insurance fund, which now needs to be reformed to enable citizens from all walks of life to access medical services.

Inadequate water, sanitation and hygiene facilities expose women and girls to violence as well as health hazards, such as gastrointestinal illnesses and respiratory and skin infections. The lack of water and sanitation also affects their ability to practice adequate menstrual hygiene, a lesser explored implication that is central to guaranteeing the well-being and equal participation of women and girls. Social disorder is on the rise as a result of the frustrations from the COVID-19 measures. Cases of risky social behavior in the community such as theft and violence have been reported in informal settlements. This comes with the increased anxiety from the economic shocks of COVID-19 and the uncertainty from the seemingly dark days ahead.

Similarly to other developing countries, in Kenya, the COVID-19 crisis has highlighted the need to address the already overburdened public health systems particularly the challenge of recruiting, deploying, retaining and protecting sufficient well-trained, supported and motivated health workers, with particular emphasis on the informal settlements [32]. Whilst a significant level of healthcare is available in some of Kenya’s large slums e.g. Kibera, there was an initial fear that COVID-19 would make the health workers sick due to lack of personal protective equipment. This situation. According to Israel Nyaburi Nyadera [18], the COVID-19 pandemic has altered the socio-economic and health dimensions of many societies across the world. For those in urban informal settlements, direct and indirect negative impacts of the pandemic and the resulting government policies have had devastating consequences on their livelihood. Racheal Nyaguthie [33] highlighted on the measures that the Government of Kenya has taken in response to the COVID-19 pandemic, “The government has recently embarked on a progressive restructuring programme to improve the highly sensitive health sector, a move that is seeing corruption cartels being edged out of the Health Ministry and key infrastructural initiatives undertaken to bring health centres closer to informal settlements”.

The Government of Kenya has earmarked the following initiatives specifically for the informal settlements in the country:

- The government is focused on medical infrastructure targeting informal settlements, with numerous projects currently underway;
- Nineteen hospitals are being built across Nairobi slums at a cost of KSh 70 million each, with another five-set to undergo rehabilitation at a cost of KSh 300 million;
- Of the 19 facilities under construction, nine will be Level Three hospitals while 10 will be Level Two hospitals; and
- The hospitals will be a huge relief to residents who have been grappling with substandard services in exploitative private clinics [33].

The slum areas that are targeted by this initiative are Viwandani, Majengo, Mathare, Kayole, Soweto, Korogocho, Kawangware, Gitare Marigu, Mukuru Kwa Njenga, Mukuru kwa Reuben, Kibera and Githurai 44. Level 3 health centres in Kenya are defined as small hospitals with minimal facilities, yet they offer services like the big hospitals. They are run by at least one doctor, clinical officers and nurses. Level 4 hospitals in Kenya include facilities that provide highly specialized services. They have the resources to offer adequate medical and surgical services [33]. In Kenya there are six different levels of health care facilities (Level 1–6). The first five are managed on the county level, the sixth level by the national government.

### Employment and income generation opportunities

Employment and business opportunities within Kenya’s informal settlements were very rapidly affected by COVID-19 from the second quarter of 2020 up to the present. The small scale and informal businesses that operate in Kenya’s informal settlements have experienced significant reduction in profits and income due to the low purchasing power of slum dwellers, as well as the reduction of business hours due to the curfew, and insecurity that has led to the closure of businesses in some slums. Furthermore, most casual labour jobs which were available to slum dwellers were drastically reduced or are no longer available. Some slum dwellers were declared redundant due to the closure of small and medium enterprises. According to the Karen Austrian and Beth Kangwana [34], within the Nairobi’s informal settlements, 39% of households reported a complete loss of income and another 48% lost part of their income.

According to Innovations for Poverty Actions [35], “While women were earning less than their male partners prior to COVID-19, this gap has been widened due to the pandemic. Half of women, compared to a third of men, report earning nothing due to coronavirus. Of those in a partnership, 44% say both they and their partner are earning less now”. According to our survey study, as a result of COVID-19, more than 40% of slum households now lack employment and their average monthly household income is 78 USD. Unfortunately, girls who normally wash clothes for the middle class who live near slum areas, have been unable to find work now because their affluent customers are afraid that the disease may spread to their homes. With no income, the situation is getting tough and girls are being taken advantage of.

Regarding the men who inhabit informal settlements the majority of whom engage in manual jobs, have had their job opportunities curtailed by COVID-19. This is because the jobs that they engage in e.g. masonry, sales, small businesses, cleaning services, hawking, boda (motor bike based transportation services), cannot be done remotely as per the government directives for all workers to consider working from their homes. This has left these precarious slum based workers with limited financial alternatives. For slum dwellers who provide non-essential night shifts, most of those jobs were stopped by the exigencies occasioned by COVID-19.

In Mombasa County, COVID-19 had a huge negative impact on tourism which saw many hotels experiencing reduced occupancy due to the pandemic restrictions. This led to huge losses in employment of slum dwellers who worked in those hotels. Elsewhere, many slum dwellers found it difficult to go out to work without violating the social distancing rule for instance in the public transport vehicles. Similarly, slum dwellers were unable to buy costly face masks and hand sanitizers due to their meagre wages [36].

However, not all persons who live in slums have been negatively affected by COVID-19 with regard to employment opportunities. Henry Owino [37] quotes Jane Atieno who is among more than 500 residents that were hired by Shofco NGO to work in 250 hand-washing stations that were established in Kibera slums. Jane Atieno admits that despite widespread job losses within informal settlements, for her, the pandemic has been a blessing in disguise. She has been earning Ksh 300 ($2.80) per day, enough money to pay all her bills.

### Questions of food security

The World Bank policy brief for Kenya of April 2020 underscores that, “Considering that the agricultural sector contributes 26% of (GDP), another 27% of GDP indirectly through linkages with other sectors, employs 40% of the total population and more than 70% of the rural population in Kenya, the impact of COVID-19 on overall economy would equally affect the agricultural and food security sector” [38]. COVID-19 pandemic coincided with start of the planting season for maize, the major staple food in Kenya. It should be noted that the early season drought caused a sharp decline in maize production in 2019 followed by the locust pandemic in early 2020, which affected food stocks leading to prices increases. A study that was conducted in 2019, covering the informal settlements of Nairobi indicated that 87% of the study households are food insecure, among which 46% are severely food insecure [39]. In total, 90% of the study households in the slums were not able to eat the kind of foods they preferred over the previous four weeks, compared to 56% for the non-slum households.

According to Elvis Mboya [23], approximately 1.3 million people in Kenya faced a crisis or worse levels of acute food insecurity, representing a decline from the estimated 2.6 million people in need of assistance in late 2019, according to the Kenya food security steering group’s 2019 short rains assessment. However, that figure was expected to increase drastically as COVID-19 pandemic penetrated the country’s food baskets in rural areas.

For slum dwellers in Kenya, sudden shocks like COVID-19 which result in disruption of income flow quickly translates into inability for such households to meet their basic needs, let alone access adequate and nutritious foods. Among the most popular coping mechanisms, include making changes to their consumption behavior, for instance, by going for cheaper but nutritionally poor diets to cater for immediate hunger needs as well as consuming less diversified diets, thus curtailing ongoing initiatives to promote consumption of adequate nutritious foods. Following COVID-19, the number of meals slum dwellers consume has decreased drastically, with many of them consuming only one meal a day. Those who would buy food especially in the evening from the street vendors are no longer able to do so. Such families end up relying on affluent well-wishers who have been regularly donating food to slum dwellers mainly in the main cities e.g. Nairobi and Mombasa. The respective county governments have also been providing food to such families.

In November 2020, Lewis Nyaundi of the Star newspaper reported that national government and the Mombasa County Government had partnered with the World Food Programme and rolled out cash transfers for 24,000 families in Mombasa County who have been adversely affected by the COVID-19 lockdown [40]. These families received Sh 4,000 per month, an amount that was thought to be enough to cover half of the monthly food and nutrition needs for a family of four persons. The cash was in addition to nutrition support for women and children. The support to poor families by the Mombasa County government was met with challenges. Masolo Mabonga [41] reported that agitated Mombasa residents accused the Governor Hassan Joho-led administration of sideling and discriminating them in the distribution of relief food and other essential goods meant for cushioning vulnerable families against the pangs of hunger caused by COVID-19 pandemic [41].

Individual NGOs have also been supporting with food security matters for the poor slum dwellers based in the informal settlements. According to Benson Ooko, a co-founder of Amani Kibera, his organization began donating foodstuffs and other items to vulnerable families after assessing the effects of COVID-19 following the introduction of the 7pm to 5am curfew across Kenya [42].

### Gender inequalities and gender-based violence

According to Agnes Odhiambo [43], “For 4 days, Juliet M., a 16-year-old Kenyan woman, was held captive by a man and sexually assaulted. The attacker reportedly said that he kidnapped her because he needed female company to get through the government-imposed COVID-19 lockdown”. This most unfortunate incident underscores the plight of many women and girls, particularly those who live in informal settlements in Kenya since March 2020.

Violence against women can have devastating consequences including sexually transmitted infections, unplanned pregnancies, HIV, physical, mental, and reproductive problems. The Kenyan government adopted strict measures to counter the spread of the COVID-19 virus, some of which have had negative impacts on women, for instance, the elevation of gender-based violence. Regrettably, the restrictions that accompanied COVID-19 made it harder for survivors to report to the authorities, as well as to seek professional help.

Victims of sexual violence in Nairobi slums have been facing a particularly difficult situation during the COVID-19 pandemic. With almost no options for transportation and a volatile situation during the night due to curfew restrictions, the number of slum dwellers who visit ‘Lavender House’, the Médecins Sans Frontières Centre dedicated to care for sexual and gender-based violence, has increased [44]. (Since 2014, the lavender-coloured building on Eastlands’ Juja Road has housed a 24/7 emergency department in addition to the longer-standing sexual violence clinic, providing cost-free care to people living precariously in the Mathare and Eastleigh slum areas [45]). For women and girls who are slum-dwellers, COVID-19 has made the challenges even greater since they face increased domestic violence and unpaid care burdens. Ordinarily in informal settlements, schools help to protect girls and young women from a lot of social injustices. But as a result of COVID-19 and the subsequent closure of schools for many months, rape and defilement cases increased with some schoolgirls engaging in prostitution just to get a source of income. According to African Population and Health Research Centre [46], there has been an increase in the cases of domestic violence and sexual assault during the COVID-19 period in Kenya. This surge could be attributed to women and young girls being trapped in with abusers by stay-at-home COVID-19 measures. The victims remain in close contact with their abusers and may not get help easily due to the disruption of services. Reports of young girls being sexually abused have become. The perpetrators of these heinous crimes are people they live with either in the same household or in the neighborhood. Most parents surveyed expressed concerns about the risk of increase in teenage pregnancies, and harassment and exploitation of women and young girls. They also expressed concerns that the economic impacts of the COVID-19 crisis on households could lead to increased domestic and gender-based violence, instability within families, and the proliferation of child prostitution and illicit sexual activities, since families cannot meet their needs [46].

According to Plan International Kenya [47], “Gender-based violence is an issue of inequality. With girls and women behind closed doors during the curfew, they are more at risk of abuse, and most of these instances of violence will go unreported. The economic impact of COVID-19 has only come to make matters worse”. Parents in slums do not have much time for their children because they must work harder to provide for their children’s basic needs. Girls who marry at a young age are at a high risk of gender-based violence, especially during the COVID-19 period. This is because most of these girls do jobs like washing clothes for other people.

### Education for slum dwellers’ children

On 15^th^ March 2020, in response to COVID-19, the Kenyan government closed all schools and colleges nationwide. This move led to the disruption of over 17 million students across the country [48]. The government reopened schools initially gradually, with some classes re-opening in October 2020 and then all classes re-opened in January 2021 [49]. In one way or another, the closure of institutions affected students and their teachers alike. For instance, many of the teachers had to look for other jobs while some of the primary schools that used rented space, had to abandon the school business altogether. School closure brought forth numerous economic and social issues, e.g. loss of learning opportunities, educational exclusion, homelessness, nutrition and economic crisis, childcare challenges and increase in teenage pregnancy cases, financial cost implication to households, and sexual exploitation. The under-privileged children and their homes based in the informal settlements felt the negative effects of COVID-19 more severely.

The government’s decision to adopt digital learning modalities for the 9 months when the schools were closed, implied that most learners, particularly those from slums, were excluded (by default) from online education due to challenges of access to internet and reliable electricity. Most slum-based parents were unable to finance their children’s school related expenses for e.g. learning materials and daily internet bundles, thereby disadvantaging their children compared to their well off counterparts who could afford these services. Essentially, this situation led to further widening the inequality gap and impeding the chances of slum-based student’s ability to access quality education and compete on a level playing field, with limited learning being one of the notable consequences within urban informal settlements. Additionally, smartphones are beyond the reach of slum dwellers. However, in slum areas that have access to electricity and the technology exists, the cost of the internet is prohibitive. Such challenges which are faced by the disadvantaged families, lead to their students being unable to compete with their more privileged fellows particularly during national examinations. Students who relied on school feeding programs found it particularly tough since their only source of food was no longer available. Historically, feeding programs have enabled students from poor families to embark on their studies without interruptions. It is worth noting that the ability of slum-based families to provide food to the children was compromised by the reduced incomes from their former jobs.

According to Catherine Jelimo [50], “Due to loss of livelihoods particularly in low-income households, some children have been forced into income-generating activities to support their families’ survival”. The hard reality is that for the majority of poor families, being able to buy food comes first before paying school fees for the children. For such families, COVID-19 led to many students taking on menial jobs, just to make ends meet. Unfortunately, some of the young girls were exploited by way of being engaged in transactional sex as a way of being able to buy essentials like sanitary towels, as well as supporting their families. Teenage pregnancies were observed to be on the increase as a result of the higher number of young girls engaging in casual labour.

### Youth living in Kenya’s slums

Measures to control COVID-19 e.g. school closures and partial lockdowns can have adverse social and economic effects on vulnerable groups. One of these groups is young people, particularly those who live in Nairobi’s low-income neighbourhoods. Results from a rapid phone-based survey [34] to collect information on knowledge, attitudes and perceived risk of infection from 1,022 adolescents living in five urban slums; Kibera, Huruma, Kariobangi, Dandora, and Mathare showed that “adolescents are experiencing great stress and anxiety due to the mitigation measures put in place. This is most likely due to school closures and the economic stress experienced by their households. People in low-income areas are starting to struggle: many are missing meals, have lost work and say that the cost of living is going up”. According to the Karen Austrian and Beth Kangwana [34], adolescents (aged 10 to 19) are in a key phase of life, making rapid transitions from childhood to adulthood. Significant negative changes, for instance, being unable to attend school or bearing children, could have long-lasting impacts on adolescents. This makes it critical for the authorities to ensure that COVID-19 does not push them beyond the point of no return.

COVID-19 has taken a toll on the mental health of adolescents in informal settlements. It appears that younger adolescents are more worried about the virus itself whereas older adolescents are more concerned about the social and economic impacts of the pandemic. The pandemic is also affecting how much adolescents eat. According to Karen Austrian and Beth Kangwana [34], of those respondents who reported skipping meals, 78% were eating less or skipping meals more often than before the COVID-19 pandemic began. This is because households had less money and the cost of food had gone up. In addition, 74% were no longer receiving the daily free meal at school that they were prior to COVID-19. Nearly half of the adolescents (46%) reported having felt down, depressed, or hopeless at least once in the past two weeks. This was more frequent the older the respondent was. The vast majority of respondents (81%) said they felt threatened, concerned, scared or anxious because of COVID-19. Most respondents (87%) also worried that they or their loved ones would be infected with COVID-19. There appears to be more pressure on boys to go out and work or look for income, while girls picked up more of the increased domestic burden—reinforcing traditional gender norms. There were small pockets of highly vulnerable adolescents that were forced by the challenges occasioned by COVID-19 to work or engage in transactional sex (receive gifts in return for sex).

### Human rights violations within Kenya’s slums

Following the imposition of a nightly curfew from 27^th^ March 2020, the human rights situation worsened as a result of widespread excessive use of force by police to enforce the curfew and other emergency measures. Documented incidents include beatings and other forms of assault; shootings; sexual violence; use of live ammunition, teargas and water cannons in residential areas, arbitrary arrests, damage to property, looting, theft and extortion. These cases included a 13-year-old boy who was shot and killed in Kiamaiko informal area based in Nairobi County, and a motorcycle taxi driver in Mombasa who took a pregnant woman to hospital after curfew and died after being beaten by police [20]. Arbitrary arrests have also been widespread, with police soliciting bribes from individuals for their release—instead of cash bail or police bond, as advised by the National Council for the Administration of Justice. In Kayole slum in Nairobi, for example, police have frequently arrested and detained civilians without recourse to the judicial system, mostly for petty offences.

In early May 2020, Kenyan government authorities evicted more than 8,000 people in two of Nairobi’s informal settlements. Deprived of their homes, hundreds of families were forced to sleep out in the open for weeks. They not only had to gather around fires for warmth but were at a higher risk of exposure to COVID-19 and of being arrested by government authorities for breaking curfews and other restrictions. The first round of evictions began on 4^th^ May 2020 in the Kariobangi North Sewerage settlement and surrounding informal settlements areas. It should be noted that the day before, a court order had ordered a halt to the impending human relocations pending a formal court hearing. However, officials from the Nairobi Water and Sewerage Company, accompanied by police, took little notice of the said court order. They arrived in the early morning and evicted about 7,000 residents, including children, from about 600 households. They also brought in excavators to demolish informal homes, churches, shops, and schools, and destroyed people’s personal belongings [51].

On 15^th^ May 2020, government authorities conducted a second round of forced evictions. This time, they arrived late at night in Ruai, another informal settlement in Nairobi, and relocated more than 1,000 slum dwellers [52]. The residents had to sleep outside in cold and rainy weather without shelter. No alternative housing was provided despite the government’s dusk-to-dawn COVID-19 curfew requiring people to stay at home or risk penalties. Regardless of the justifications, these two evictions are outrageous particularly given the attendant COVID-19 when social distancing and access to water are crucial to protecting public health. Access to adequate housing is critical to protect people from the COVID-19 infection, prevent its spread and allow those infected to recover. Moreover, these evictions do not meet standards set by both Kenyan and international law. Regarding the 4^th^ May 2020 removals, for instance, officials say verbal notice was given two days prior. However, Kenya’s 2009 eviction and resettlement guidelines require notice to be written or published in the official government gazette 90 days ahead of the actual evictions [52].

Forced evictions cause severe trauma, reduce a community’s standard of living, and worsen the economic situation of vulnerable and marginalized groups like slum dwellers. At their core, the forced relocations violate basic human rights, chiefly the right to adequate housing, irrespective of the type of “ownership” residents have. The forced human relocation also interfere with other rights such as to food, water, health, property, security of the home, and freedom from cruel, inhumane, and degrading treatment.

## Conclusion and recommendations

We can conclude that the human rights situation worsened as a result of widespread excessive use of force by police to enforce the curfew and other emergency measures. Documented incidents included beatings and other forms of assault; shootings; sexual violence; use of live ammunition, teargas and water cannons in residential areas, arbitrary arrests, damage to property, looting, theft and extortion.

It is highly likely that some of the COVID-19 response measures particularly curfews, lockdowns and containment, have highlighted pre-existing deep social inequalities in Kenya which have resulted in many slum dwellers being pushed further into poverty.

People in slum areas in Kenya may be at a higher risk of contracting COVID-19 due to overcrowding, limited access to running water and employment opportunities to cater for their needs.

For slum dwellers, government restriction measures also have had detrimental effects on the mental health and socio-economic aspects of individuals, care givers and communities.

Due to loss of livelihoods particularly in low-income households, some children have been forced into income-generating activities to support their families’ survival.

In light of all these, it is recommended that governmental steps are taken about the following:

- to educate law enforcement agencies on how to use non-violent methods of managing pandemics like COVID-19;
- to address the social and economic disparities that have been exposed by COVID-19, particularly with regard to slum dwellers;
- to enhance governmental programmes that address slum dwellers given their precarious situation;
- to prioritize mental health as a serious challenge that has been accelerated by COVID-19, particularly for slum dwellers; and
- to make concerted efforts aimed at enabling the children from low-income households, most of which are found in slums, to enable them to resume learning.

## Supporting information

S1 File Questionnaire_ for “The impact of Covid-19 on the livehoods of Kenyan slum dwellers”. (PDF)

S2 File Inclusivity in global research policy. (DOCX)
