# Supplementary material for: The impact of COVID-19 on the livelihoods of Kenyan slum dwellers and the need for an integrated policy approach
Source: PLoS One. 2022 Aug 2;17(8):e0271196. doi: 10.1371/journal.pone.0271196 (PMC9345334; doi:10.1371/journal.pone.0271196)
Supplement: S1 File — (PDF) [file pone.0271196.s001.pdf]

QUESTIONNAIRE FOR "THE IMPACT OF COVID-19 ON THE LIVEHOODS OF KENYAN SLUM DWELLERS"

INTRODUCTION AND INFORMED CONSENT

Good morning/afternoon/evening. Thank you for the opportunity to speak to us. Our names are Daniel Solymari and Edward Kairu. Please note that we might have some research assistants who will ask the questions on our behalf. We are independent researchers who are interested in the informal settlements of Kenya. Today we are carrying out a short face to face survey among randomly selected slum dwellers in Kenya particularly to find out the impact of COVID-19 on their livelihoods. You are one of the leaders of the slums that we have randomly selected to participate in the survey.

We would like to ask you a few questions concerning your community's experiences with COVID-19 pandemic particularly on how it has impacted the residents of the slum village that you represent. Your responses will help decision makers here in Kenya and elsewhere make the useful interventions with a view to improving the livelihoods of slum dwellers her in Kenya

We will first explain what we are going to do the interview.

Procedures: If you are willing and accept, we will ask questions on the impact of COVID-19 on the different aspects of the livelihoods of your Community Based Organization's members. This interview will take about 15 – 20 minutes of your time.

Risks: We will take precautions to keep any information you give us during the interview confidential. For example, your name or other identifying information will not appear on any of our records of responses. During the interview, you can decline to answer any particular question, or stop the interview at any point. Your responses will be available only to the team conducting this study. We intend to do this interview in private; if someone approaches us, we may stop the interview until we can continue in private.

Benefits: There are no direct and immediate benefits to you for participating in this interview. In some exceptional cases, there may be indirect benefits to yourself or the Community Based Organization that you represent, by way of projects being implemented in your slum by development agencies here in Kenya, as a result of the paper that we shall publish.

Confidentiality: At the end of the study, we will put all the answers together and write a professional paper that we shall submit to credible journals. We will not identify you by name, or the slum that you represent, in our journal publication. Your responses to this interview will be seen only by the researchers, and will be stored in a locked place under our control.

Compensation: You will not receive money for participating in the interviews and any related training.

Voluntary Participation: Taking part in this study is completely voluntary. If you choose to take part, you may stop at any time or skip any questions that you do not want to answer. Please note that your choice to take part in this interview or not to take part in this interview will in no way affect or hinder your livelihood, or your participation in future activities were the paper that we plan to have published, lead to a development project implemented in your slum. If you have any questions or concerns about taking part in this study, please feel free to talk to us and we shall be happy to answer your questions to the best of our abilities. You can also ask questions at any time about the paper that we plan to have published. You can take this consent form with you if you want to review it further.

Persons to Contact: If you want to talk to anyone about this study because you think you have not been treated fairly or think you have been harmed in any way by joining the study, or you have any other questions about the study in the future, you may speak directly with the principal researchers who are:

- Daniel Solymari solymari.daniel@maltai.hu
- Edward Kairu edward.kairu36@gmail.com

We will leave one copy of this form with you so that you will have record of this contact information and about the study.

* * *

I certify that I have read discussed the consent procedures above with the interviewee/participant and continued only on his/her consent.

Name of Enumerator: ________________________

Signed: ______________________________ Date: ______________

THANK RESPONDENT THEN BEGIN THE INTERVIEW

A	BACKGROUND								
**No.**	**Question**	**Response**							
	Start time								
**A2**	Date of interview	**D**	**D**	**M**	**M**	**2**	**0**	**2**	**1**
**A3**	Operator ID								
**A4**	Interviewed by (Name)								
**A5**	Respondent's name and CBO he/she represents								
**A6**	Respondent's contact phone number								
**A7**	Respondent Sex	**1 = Male; 2 = Female**							
**A8**	Respondent's status in the family	______________________________ ☐ Less than 15 ☐ 15-29Yrs ☐ 30-35Yrs ☐ Above 35 Yrs							
**A9**	Number of children in the family	**1 = Male** **2 = Female**							
**A10**	County name								
**A11**	Sub county name								
**A12**	For how long have you lived in this slum								
**A13**	Physical Address (optional)								

Questions for each respondent

1. What is the name of this slum in which the CBO that you represent is located?
2. When was that slum established?
3. How many women, men, children live in this slum?
4. Before the onset of COVID-19, what was the general life like in this slum?
5. How would you rate the kind of water and sanitation services that are available in this slum?
6. How has the government of Kenya's response to COVID-19 been like in this slum?
7. Looking at the youth in your slum, what would say at the most significant negative impacts of COVID-19 to-date?
8. Regarding employment, can you indicate the percentages of the slum dwellers that you represent who have lost their jobs due to COVID-19 pandemic?
9. To what extent have the slum dwellers in the slum that you represent been able to comply with the guidelines that have been issued by the Ministry of Health with a view to reducing the impact of COVID -19?
10. In the past, how many NGOs implemented projects in this slum?
11. Since COVID-19 was observed in Kenya in March 2020, how many new NGOs have implemented projects in this village?
12. Which are the kind of interventions that have been implemented by NGOs in this slum?
13. Which are the most effective NGOs that have benefited most of the slum dwellers in your area of jurisdiction?
14. How has COVID-19 impacted on the small scale activities that are normally carried out by slum dwellers in your slum?
15. How has COVID-19 impacted on food security in the slum that you represent?
16. How has COVID-19 impacted on gender roles and inequality in the slum that you represent?
17. How has COVID-19 impacted education of the children that live in the slum that you represent?
18. How has COVID-19 impacted on the human rights of the persons who live in this slum that you represent?
19. Are there any recommendations that you would like to offer for any development agencies that might consider implementing projects in the slum that you represent?
20. Do you have any question that you would like to be answered?
