# Supplementary material for: The impact of COVID-19 on the livelihoods of Kenyan slum dwellers and the need for an integrated policy approach
Source: PLoS One. 2022 Aug 2;17(8):e0271196. doi: 10.1371/journal.pone.0271196 (PMC9345334; doi:10.1371/journal.pone.0271196)
Supplement: S2 File — (DOCX) [file pone.0271196.s002.docx]

Inclusivity in global research

PLOS’ policy on inclusivity in global research aims to improve transparency in the reporting of research performed outside of researchers’ own country or community and ensures that PLOS publications reporting global research adhere to high standards for research ethics and authorship. Authors of relevant research articles may be asked to complete the questionnaire below, which outlines ethical, cultural, and scientific considerations specific to inclusivity in global research. This questionnaire may be requested when researchers have travelled to a different country to conduct research, if research uses samples collected in another country, research with Indigenous populations or their lands, or if research is on cultural artefacts. Researchers travelling to another country solely to use laboratory equipment will not normally be required to complete the questionnaire. However, the questionnaire can be requested at the journal’s discretion for any submission – if you have been requested to complete this questionnaire by the PLOS journal you submitted to, please do so.

Please complete the questionnaire below and include this as a Supporting Information file with your manuscript. Note that if your paper is accepted for publication, this checklist will be published with your article in the supporting information files. Please ensure that you reference the checklist in the main body of your manuscript. We suggest adding a subsection ‘Inclusivity in global research’ to your Methods section and adding the following sentence: “Additional information regarding the ethical, cultural, and scientific considerations specific to inclusivity in global research is included in the Supporting Information (SX Checklist)”

The questions have been designed to be applicable to a wide range of study types, and there are subsections for both human subjects research and non-human subjects research. If any of the questions are not relevant to your research please mark them as “N/A” as appropriate.

**Ethical considerations, permits and authorship**

*This section is applicable to all research types.*

Provide details as to who granted permissions and/or consent for the study to take place in the Methods section of your manuscript. This should include the names of **all** ethics boards, governmental organizations, community leaders or other bodies that provided approval for the study. If individuals provided approval refer to these people by their role or title but do not list their name(s).

Reported on page number: 10

The ethical approval of the study was designed as per (1) the Humanitarian Charter of the Sphere Project and Handbook in line with the CHS Humanitarian Minimum Standards, (2) the Code of Conduct of the Hungarian Charity Service of the Order of Malta and its CEO and (3) the Scientific Research Ethics Committee of the Medical Research Council of Hungary. The latter one is based on the Codex of Ethics of Scientific Knowledge of the Hungarian Academy of Sciences. The study had been reported to the State Department of Housing and Urban Development in Nairobi, therefore, it was in line with the national regulation and law applying to researchers.

If there were any deviations from the study protocol after approval was obtained please provide details of these changes in the Methods section of your manuscript.
Did this study involve local collaborators that are residents of the country where the research was conducted or members of the community studied? If you do not have any authors from said communities, please provide an explanation for this below.

The study involved local collaborators that are residents of the country. Based upon the required ethics approvals, as well as consent to participate procedure, we contacted the government and local NGO actors active in the selected slums and requested their assistance in conducting the interviews. Consequently, the interviews were conducted with the help of local assistants (local leaders, social workers, co-workers of NGOs, etc.) who were familiar with the respective informal urban settlement.

Reported on page number: N/A

Everyone listed as an author should meet PLOS’ criteria for authorship and all individuals who meet these criteria should be included in the author byline, rather than the acknowledgements. Authorship criteria is based on the International Committee of Medical Journal Editors (ICMJE) Uniform Requirements for Manuscripts Submitted to Biomedical Journals - for further information please see here: <https://journals.plos.org/plosone/s/authorship>.

**Human subjects research (e.g. health research, medical research, cross-cultural psychology)**

Did you obtain written informed consent from a representative of the local community or region before the research took place? How did you establish who speaks for the community? Details of written informed consent obtained from study participants should be reported separately in the Methods section of your manuscript.

How did members of the local community provide input on the aims of the research investigation, its methodology, and its anticipated outcome(s)?

Members of the local community can be divided into two groups based on their input on the research investigation:

1. those who participated on the intervewer,
2. and those who participated on the interviewee side.

Based upon the required ethics approvals, as well as consent to participate procedure, the interviews were conducted with the help of local assistants (local leaders, social workers, co-workers of NGOs, etc.) who were familiar with the respective informal urban settlement. For their part, the input was their free time, local knowledge and familiarity, which increased the willingness to respond among the surveyed community.

Those community members who participated on the interviewee side, their answers were the input, which provided useful, comprehensive information for the research.

On the national level the study had been reported to the State Department of Housing and Urban Development in Nairobi, therefore, it was in line with the national regulation and law applying to researchers.

On the local level it is important to highlight that, first of all, personal participation in connection with the study happened on a voluntary basis (see page number: 10 and 13). The respondents were selected by random sampling methodology (see page number 12 and 13).

Secondly, in order to get the full consent of the examined communities, the interviews were conducted with the help of local assistants (local leaders, social workers, co-workers of NGOs, etc.) who were familiar with the respective informal urban settlement.

Whatismore, community members who chose to answer the interview questions were first read a *Consent to participate in research* form by the interviewer, which provided detailed information about the research - including the purpose of the research, what kind of questions they could expect to be asked, how will the confidentiality of the research records be protected, and who they could contact if they had further questions etc. As part of the information it was stated that

1. the interview is anonymous,
2. participation in this study is voluntary and the respondant can choose not to participate without any repercussions,
3. information this study will be kept confidential.

The consent of the respondent was not given in writing but verbally.

When engaging with the local community, how did you ensure that the informed consent documents and other materials could be understood by local stakeholders?

As the interviews were conducted with the help of local assistants (local leaders, social workers, co-workers of NGOs, etc.) who were familiar with the respective informal urban settlement, their familiarity with the place, and their language knowledge was the guarantee that the *Consent to participate in research* form, and all other relevant information about the research was fully understood by the local stakeholders.

Will the findings of the research be made available in an understandable format to stakeholders in the community where the study was conducted (e.g. via a presentation, summary report, copies of publications, etc.)? Please provide details of how this will be achieved.

Since based on the methodology of the research, the respondents of interviews were randomly selected pedestrian, the direct feedback of the research result is not possible. However, a summary of the findings will be sent to each and every local assistnats we have contacted.

**Non-human subjects research using specimens/ animals collected as part of the study, or those housed in archival collections. Examples include archaeology, paleontology, botany and zoology.**

Did the permission you obtained from a local authority to perform the study include an agreement on access to outputs and benefit sharing? This may include procedures to enable fair distribution of the benefits and resources arising from the research performed. Please include any details of Prior Informed Consent and Benefit Sharing Agreements obtained. These may be required by field-specific regulations, for example the Convention on Biological Diversity (CBD) and the associated Nagoya Protocol.

If the material used in your study was imported, please A) provide the year it was imported and B) indicate whether permits were obtained to import/export the materials used, C) provide details of any permits obtained. If this information is not available, please indicate this.

If you used archival specimens, please state how the material used in your study was acquired by the institute it is held in and provide details of any permits obtained for the original excavations/ sample collection. If this information is not available, please indicate this.

How was the potential cultural significance of the materials collected in your study to local communities considered in your research design? Were Indigenous peoples and/or local researchers and institutions involved with archaeological excavations / collection of specimens? If so, please provide a description of their involvement.

If your manuscript includes photographs of human remains please indicate whether authors obtained permission from descendants or affiliated cultural communities to do so.
